# A radiohybrid theranostics ligand labeled with fluorine-18 and lutetium-177 for fibroblast activation protein-targeted imaging and radionuclide therapy

**DOI:** 10.1007/s00259-023-06169-5

**Published:** 2023-03-03

**Authors:** Tianhong Yang, Lei Peng, Jia Qiu, Xingjin He, Dake Zhang, Renbo Wu, Jianbo Liu, Xiangsong Zhang, Zhihao Zha

**Affiliations:** Department of Nuclear Medicine, The First Affiliated Hospital of Sun Yat-Sen University, #58 Zhongshan Er Road, Guangzhou, 510080 Guangdong Province China

**Keywords:** Silicon-fluoride-acceptor, FAP inhibitor, Radiohybrid, Theranostics

## Abstract

**Purpose:**

A series of radiotracers targeting fibroblast activation protein (FAP) with great pharmacokinetics have been developed for cancer diagnosis and therapy. Nevertheless, the use of dominant PET tracers, gallium-68–labeled FAPI derivatives, was limited by the short nuclide half-life and production scale, and the therapeutic tracers exhibited rapid clearance and insufficient tumor retention. In this study, we developed a FAP targeting ligand, LuFL, containing organosilicon-based fluoride acceptor (SiFA) and DOTAGA chelator, capable of labeling fluorine-18 and lutetium-177 in one molecular with simple and highly efficient labeling procedure, to achieve cancer theranostics.

**Methods:**

The precursor LuFL (**20**) and [^nat^Lu]Lu-LuFL (**21**) were successfully synthesized and labeled with fluorine-18 and lutetium-177 using a simple procedure. A series of cellular assays were performed to characterize the binding affinity and FAP specificity. PET imaging, SPECT imaging, and biodistribution studies were conducted to evaluate pharmacokinetics in HT-1080-FAP tumor-bearing nude mice. A comparison study of [^177^Lu]Lu-LuFL ([^177^Lu]**21**) and [^177^Lu]Lu-FAPI-04 was carried out in HT-1080-FAP xenografts to determine the cancer therapeutic efficacy.

**Results:**

LuFL (**20**) and [^nat^Lu]Lu-LuFL (**21**) demonstrated excellent binding affinity towards FAP (IC_50_: 2.29 ± 1.12 nM and 2.53 ± 1.87 nM), compared to that of FAPI-04 (IC_50_: 6.69 ± 0.88 nM). In vitro cellular studies showed that ^18^F-/^177^Lu-labeled **21** displayed high specific uptake and internalization in HT-1080-FAP cells. Micro-PET, SPECT imaging and biodistribution studies with [^18^F]/[^177^Lu]**21** revealed higher tumor uptake and longer tumor retention than those of [^68^ Ga]/[^177^Lu]Ga/Lu-FAPI-04. The radionuclide therapy studies showed significantly greater inhibition of tumor growth for the [^177^Lu]**21** group, than for the control group and the [^177^Lu]Lu-FAPI-04 group.

**Conclusion:**

The novel FAPI-based radiotracer containing SiFA and DOTAGA was developed as a theranostics radiopharmaceutical with simple and short labeling process, and showed promising properties including higher cellular uptake, better FAP binding affinity, higher tumor uptake and prolong retention compared to FAPI-04. Preliminary experiments with ^18^F- and ^177^Lu-labeled **21** showed promising tumor imaging properties and favorable anti-tumor efficacy.

**Supplementary Information:**

The online version contains supplementary material available at 10.1007/s00259-023-06169-5.

## Introduction

Fibroblast activation protein (FAP), a type II transmembrane serine protease of the prolyl oligopeptidase family, is commonly expressed in cancer-associated fibroblasts (CAFs). FAP-positive CAF subpopulations accumulate in cancers with poor prognosis and are linked to tumor progression and immunosuppression. More importantly, FAP-positive CAFs are often abundant in the tumor microenvironment in more than 90% of epithelial carcinomas, while the FAP expression in normal tissues is usually undetectable [1], thereby making FAP a promising pan-tumor target for cancer diagnosis and therapy [1, 2].

One approach to utilize FAP in cancer treatment is through radioisotopes-labeled theranostics ligands. Since the first FAP-specific inhibitor (FAPI)–based radioligand FAPI-04 was reported in 2018 [3], a number of quinoline derivatives, including FAPI-02, FAPI-21 and FAPI-46, have been developed and showed excellent diagnosis outcomes [4]. These tracers could be labeled with gallium-68 and provide advantages over [^18^F]FDG in certain tumors [5–7]. Although these ligands demonstrated encouraging results in cancer diagnosis, radionuclide therapy with ^177^Lu-labeled FAPI-04 or FAPI-46 are still hindered by their rapid clearance from circulation and inadequate tumor accumulation [8, 9]. Subsequent structure–activity relationship studies to improve pharmacokinetics of FAPI-based ligands resulted in fatty acid/albumin binder-conjugated tracers and bivalent FAPI ligand [10–12]. Preclinical studies with tumor bearing mice indicated favorable anti-tumor responses. It is noteworthy that the FAPI-based radiotracers were mainly established around highly potent FAP inhibitor UAMC1110 [13]. Meanwhile, a novel class of FAP-targeting radiopharmaceutical, FAP-2286, was developed utilizing cyclic peptides as binding motif [14]. Nevertheless, further clinical translation studies are warranted to validate the efficacy of these new tracers in the treatment of FAP positive tumors. Additional therapeutic radioligands with improved pharmacokinetics are urgently needed for cancer treatment [15].

FAP-targeted PET imaging agents are commonly labeled with gallium-68 [3–7]. Nevertheless, fluorine-18 is still the most widely used positron emitting isotope in PET imaging due to the superior physico-chemical properties [16]. Consequently, ^18^F-labeled FAPI analogs have been developed with great effort, and [^18^F]FGlc-FAPI is a case in point [17]. However, [^18^F]FGlc-FAPI was produced in time-consuming and multi-step radiosynthesis procedure, which hampered the clinical translation.

Recently, to simplify the labeling technologies that introduce fluorine-18 into large and complex organic compounds, several highly efficient and reliable radiosynthesis methodologies have been developed, such as silicon-^18^F, boron-^18^F, and aluminum-^18^F [18]. Among these procedures, aluminum-^18^F has been successfully applied in FAP-targeting imaging agents, leading to the establishment of [^18^F]FAPI-74 [16] and [^18^F]AlF-P-FAPI [19]. In particular, silicon-^18^F methodology that exploited silicon-fluoride-acceptor (SiFA) approach showed more favorable chemical kinetics [20]. The isotopic exchange of ^18^F-^19^F could be done in 5–10 min, at room temperature (r.t.) with high radiochemical yields (RCY) and high molar activities. Several radioligands implementing this strategy have been validated in clinical translation, including [^18^F]SiTATE [21] and [^18^F]rhPSMA [22].

In this study, we developed a novel FAPI analog, LuFL, that contains SiFA and DOTAGA moieties in a single molecule. This radiohybrid FAP-targeting ligand could be labeled with fluorine-18 through SiFA isotopic exchange, while the chelator DOTAGA was able to coordinate with lutetium-177 for radionuclide therapy. Additional structural modification by introducing glycine and tranexamic acid was made to adjust the lipophilicity and improve the pharmacokinetics. Herein, we present the synthesis, radiolabeling and characterization of [^18^F][^nat^Lu]Lu-LuFL ([^18^F]**21**) and [^177^Lu][^nat^F]Lu-LuFL ([^177^Lu]**21**) for FAP-targeting PET imaging and radionuclide therapy.

## Materials and methods

### Synthesis and radionuclide

All reagents were commercially available and used without further purification unless otherwise indicated. The synthesis route of compound LuFL (**20**) [13, 23, 24] and [^nat^Lu]Lu-LuFL (**21**) were described in detail in the Supplementary Information (Supplementary Scheme 1). No-carrier-added [^18^F]F^−^ was acquired from an in-house PETtrace cyclotron (GE Healthcare). The solution of [^177^Lu]LuCl_3_ (3.7 GBq, 100 μL, in 0.04 M HCl) was kindly provided by Chengdu Gaotong Isotope Co., Ltd. [^68^ Ga]GaCl_3_ was eluted with 0.05 N HCl from ^*68*^*Ge*/^*68*^* Ga* generator (ITG, Germany).

### Radiolabeling

The procedure for the radiosynthesis of [^18^F]**21** was similar as the method described previously [20]. Briefly, aqueous [^18^F]F^−^ (125.8–7400 MBq) was trapped on a Sep-Pak Plus QMA and eluted with [K^+^2.2.2]OH^−^ solution. Oxalic acid and **21** (30 μL or 75 μL, 1 mg/mL, in DMSO) were then added to the eluate. After maintaining at r.t. for 5 min, the mixture was diluted with PBS and purified by HLB cartridge to obtain [^18^F]**21**.

For labeling of [^177^Lu]**21**, to a solution of **20** (10 μL, 1 mg/mL in DMSO) NaOAc (15 μL, 2 M) and [^177^Lu]LuCl_3_ solution (74–259 MBq, 0.5 mL, in 0.05 M HCl) was added. The mixture was heated at 90 ℃ for 10 min and the resulting [^177^Lu]**21** was used without further purification.

For labeling of [^68^ Ga]Ga-FAPI-04 or [^177^Lu]Lu-FAPI-04, [^68^ Ga]GaCl_3_ (70–111 MBq, 0.443 mL, in 0.05 N HCl) or [^177^Lu]LuCl_3_ solution (70–273 MBq, 0.443 mL, in 0.05 N HCl) was added into the FAPI-04 (7 μL, 1 mg/mL, in DMSO), followed by sodium acetate solution (50 μL, 0.5 N). The mixture was heated at 90℃ for 10 min to obtain the [^68^ Ga]Ga-FAPI-04 or [^177^Lu]Lu-FAPI-04.

### In vivo* and *in vitro* stability*

[^18^F]**21** or [^177^Lu]**21** (50 μCi, 10 μL) was added to PBS (90 μL) or murine serum (90 μL) and the mixture was incubated at 37 °C for a certain time ([^18^F]**21**: 2 h, [^177^Lu]**21**: 8 d). The PBS mixture was injected directly into the radio-HPLC for analysis. The murine serum was precipitated with 0.1 mL of acetonitrile and centrifuged (10,000 rpm, 5 min). The supernatant was measured and analyzed by the radio-HPLC to determine the stability. For in vivo stability study, the radio tracers were injected into the tail vein of ICR mice and samples of blood were collected after a certain time post injection (pi) ([^18^F]**21**: 4 h, [^177^Lu]**21**: 9 h). The plasma proteins were precipitated using an equal volume of acetonitrile and centrifuged (10,000 rpm, 5 min). The RCP of radio tracer in supernatants were analyzed and quantified by radio-HPLC. The radio-HPLC method was as follows: A: 0.1% TFA in H_2_O, B: ACN, 0–10 min, 0–100% B. The flow rate was 1 mL/min, and the C18 column (4.6 × 150 mm, 5 μm, ZORBAX, Agilent) was used. When using HPLC to assess the stability of [^177^Lu]**21**, the effluent was collected every 30 s due to its less radioactivity, and samples were measured by an automatic γ-counter (2480 Wizard2, Perkin Elmer). The counted samples were plotted as intensity (cpm) against fraction [19].

### Partition coefficient

The radiotracers were added to equal volumes of n-octanol and PBS buffer, vortexed for 2 min at room temperature. Then, the mixture was centrifuged and equal volumes of n-octanol and PBS solutions were taken separately to be counted by an automatic γ-counter, and the average LogP value was calculated.

### Cellular assays

The human fibrosarcoma cell lines HT-1080 and HT-1080-FAP transferred with human FAP gene were both cultured in Dulbecco modified Eagle medium supplemented with 10% fetal bovine serum (Gibco, USA) at 37℃/5% carbon dioxide. For cell uptake experiments, HT-1080-FAP cells (~ 1 × 10^5^/well) or HT-1080 (~ 1 × 10^5^/well) were seeded in 24-well plates for 24–48 h and incubated at 37℃ with radiolabeled compounds (74 kBq/well) in 0.5 mL of serum-free medium for indicated times. Non-specific binding was determined by co-incubating with 100 μM FAP inhibitor UAMC1110. For competitive FAP binding assays, HT-1080-FAP cells were incubated with [^177^Lu]Lu-FAPI-04 (74 kBq/well) in the presence of different concentrations (10^−3^–10^4^ nM) of competing non-radioactive ligands. For internalization experiments, the radiotracer-incubated cells were washed twice with PBS (pH 7.4), followed by glycine–HCl (0,05 M, pH = 2.8) solution to distinguish between cell surface–bound (acid-releasable) and internalized (acid-resistant) radioligand. For efflux experiments, cells were preincubated with [^177^Lu]**21** or [^177^Lu]Lu-FAPI-04 for 1 h and subsequently incubated at 37 °C in a medium free of radiotracer and serum for different time periods. The medium was removed and the cells were washed twice with cold PBS (pH 7.4) and subsequently lysed with 0.5 mL of NaOH (1 M). Cell lysates were collected and the radioactivity was determined using a γ-counter.

### Preparation of xenograft models

All animal experiments were conducted in compliance with a protocol approved by the First Affiliated Hospital of Sun Yat-sen University Institutional Animal Care and Use Committee. BALB/C-nu/nu mice (4–5-week old with body weight of 16–22 g) were purchased from GemPharmatech Co., Ltd. Mice were housed under standard conditions with temperature and light control (12-h-light/12-h-dark cycle) and had free access to water and food. For the tumor model, HT-1080-FAP or HT-1080 cells (5 × 10^6^) were subcutaneously injected into the right or left flank of nude mice, respectively. As the tumor diameter reached 3–4 mm, mice were treated with radioligands. Micro-PET and SPECT imaging studies, as well as biodistribution, were conducted when the tumor diameter reached 5–10 mm.

### Micro-PET/CT imaging

The dynamic micro-PET/CT (Siemens Inveon) imaging studies were conducted to evaluate the pharmacokinetics. The HT-1080-FAP and HT-1080 tumor-bearing mice were injected with 3.7–7.4 MBq of [^18^F]**21** or [^68^ Ga]Ga-FAPI-04 via tail vein. For blocking studies, FAPI-04 (25 μg/animal, *n* = 3) was co-injected with [^18^F]**21** (3.7–7.4 MBq), and the whole body scan was performed at 60 min post-injection (pi). Dynamic PET scans were performed from 0 to 120 min with image reconstruction of every 5 min. Micro-PET imaging was reconstructed using a three-dimensional ordered-subset expectation maximum (OSEM) algorithm (Siemens, Erlangen, Germany). The images and regions of interest (ROIs) were produced using Inveon Research Workplace (Siemens, Erlangen, Germany).

### SPECT imaging

The mice bearing HT-1080-FAP or HT-1080 xenografts were injected with about 37 MBq of [^177^Lu]**21** via the tail vein, and SPECT (Siemens, Symbia Intevo Bold, Germany) was performed at 2 h to 8 d after injection. The SPECT parameters were as follows: medium-energy collimator, energy peaks of 113 and 208 keV, matrix of 256 × 256, and scan duration of 10 min.

### Biodistribution studies

[^177^Lu]**21** (~ 0.925 MBq) was injected via the tail vein of HT-1080-FAP tumor-bearing mice. The animals were sacrificed at 4, 24, 48 and 96 h pi. Organs of interest were dissected, weighed and counted with a γ-counter. The data were normalized to percentage injected dose (%ID/g) with standard dilution of the initial dose. The biodistribution of [^177^Lu]Lu-FAPI-04 was conducted at 24 h pi for comparison.

### Radionuclide treatment experiments

Radionuclide therapy was performed when the diameter of HT-1080-FAP xenografts reached 3–4 mm. The mice were randomly divided into 4 groups (*n* = 4) and intravenously injected with [^177^Lu]**21** (24.05 MBq), [^177^Lu]**21** (9.25 MBq), [^177^Lu]Lu-FAPI-04 (24.05 MBq) and saline, respectively. Tumor volume (volume = length × width^2^/2) and mouse weight were measured every 2–3 d. Endpoint criteria were defined as weight loss of more than 15%, tumor volume greater than 1500 mm^3^, or active ulcerations of the tumor. After treatment, the major organs of the mice were removed for hematoxylin and eosin (H&E) staining following the previous study [11]. Immunohistochemical staining of tumors was performed with Anti-human FAPmAb (Abcam, ab207178).

### Autoradiography and histology

The slides of HT-1080-FAP tumor were incubated with [^18^F]**21** for 1 h, exposed on a phosphoscreen, and subsequently scanned on a Beckman Coulter FLA7000IP Typhoon storage phosphorimager. Immunohistochemical staining of FAP was performed on the adjacent section.

### Statistical analysis

Data were presented as mean ± SD using the Origin Pro 2021 (OriginLab Corporation) and GraphPad Prism 8 (GraphPad Software). IBM SPSS statistics 21 (IBM Corporation) was used to analyze the data and differences between groups were compared using ANOVA or *t*-test. *P*-values < 0.05 were considered statistically significant.

## Results

### Synthesis and radiochemistry

**Fig. 1:**
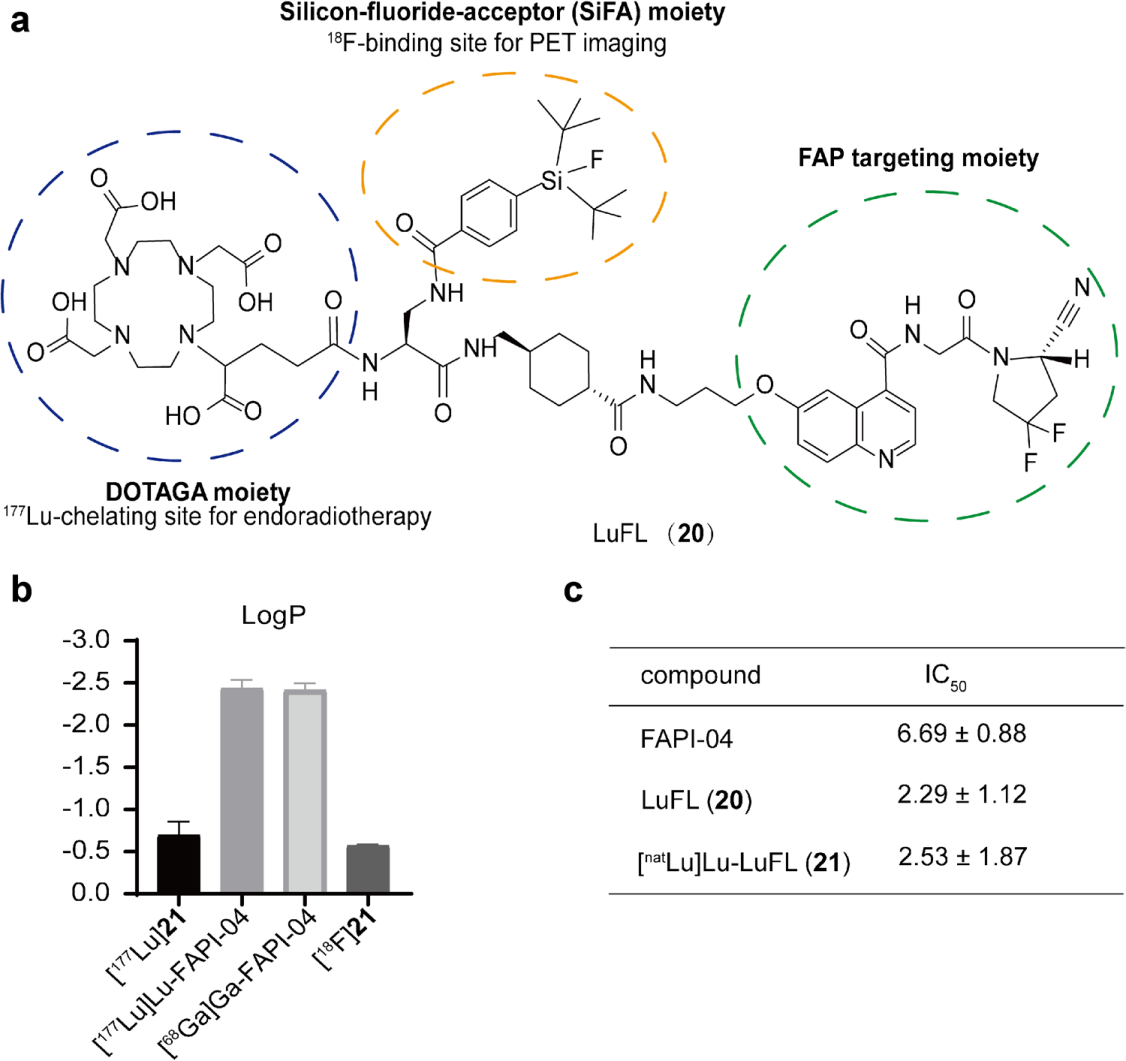
Chemical structure of **20** (**a**). LogP of radiopharmaceuticals (**b**). IC_50_ of compounds FAPI-04, LuFL (**20**), [^nat^Lu]Lu-LuFL (**21**) (**c**)

The precursor (**20**) was successfully synthesized (Fig. 1a). Due to the simplicity of one-step ^18^F-^19^F isotope exchange reaction and the absence of chemical side products, [^18^F]**21** was obtained in 20 min with a RCY of 18.1–61.8% (*n* = 10). The molar activities of [^18^F]**21** was 1.17–106 GBq/μmol. For the labeling of [^177^Lu]**21**, the decay corrected RCY was over 99% and the molar activities were 9.5–37.1 GBq/μmol.

Both [^18^F]**21** and [^177^Lu]**21** showed excellent in vitro and in vivo stability (Supplementary Fig. 1). Results suggested that [^18^F]**21** was stable for at least 2 h in vitro, whereas [^177^Lu]**21** maintained stable after incubating in saline and serum for 8 days (RCP > 90%). Radio-HPLC analyses of blood samples displayed little evidence of any metabolization of the ligands, [^18^F]**21** and [^177^Lu]**21**, within a period of 4 and 9 h, respectively. The LogP (Fig. 1b) of [^18^F]**21** and [^177^Lu]**21** was − 0.57 ± 0.02 and − 0.69 ± 0.13, respectively, indicating that the tracers were more lipophilic than [^68^ Ga]Ga-FAPI-04 (LogP: − 2.42 ± 0.08) and [^177^Lu]Lu-FAPI-04 (LogP: − 2.44 ± 0.10).

### In vitro* evaluation*

**Fig. 2:**
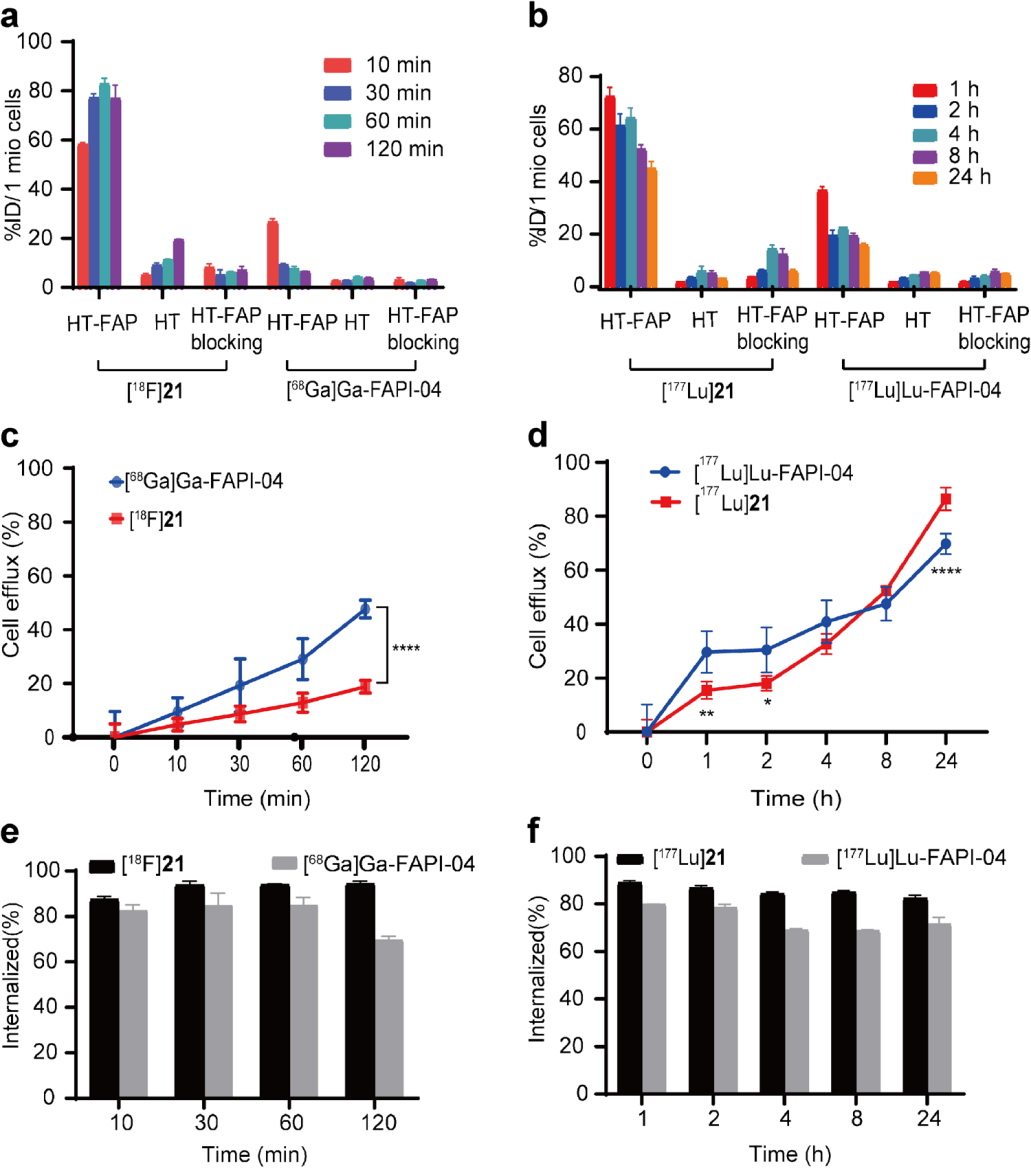
In vitro characterization of FAPI radioligands. Cell uptake assays of [^18^F]**21** (**a**) or [^177^Lu]**21** (**b**) in HT-1080-FAP (FAP positive) and HT-1080 (FAP negative) cells compared with [^68^ Ga]Ga-FAPI-04 (**a**) or [^177^Lu]Lu-FAPI-04 (**b**), respectively, with and without the competitor UAMC1110. The efflux of [^18^F]**21** (**c**) or [^177^Lu]**21** (**d**) in HT-1080-FAP cells compared with [^68^ Ga]Ga-FAPI-04 (**c**) or [^177^Lu]Lu-FAPI-04 (**d**), respectively. The internalization of [^18^F]**21** (**e**) or [^177^Lu]**21** (**f**) in HT-1080-FAP cells compared with [^68^ Ga]Ga-FAPI-04 (**e**) or [^177^Lu]Lu-FAPI-04 (**f**), respectively. HT-FAP = HT-1080-FAP. HT = HT-1080. *P < 0.05. **P < 0.01. ****P < 0.0001

Compound **20** and **21** displayed significantly higher binding affinity than that of FAPI-04 (Fig. 1c, IC_50_: 2.29 nM and 2.53 nM vs. 6.69 nM). [^18^F]**21**/[^177^Lu]**21** showed significantly higher uptakes in HT-1080-FAP cells than those of [^68^ Ga]/[^177^Lu]Ga/Lu-FAPI-04 at all time points (e.g. [^18^F]**21** vs. [^68^ Ga]Ga-FAPI-04: 86.43% ± 5.47% vs. 7.65% ± 1.00%, 1 h), and the uptakes could be blocked by FAP inhibitor. Almost no uptakes in HT-1080 cells were observed (Fig. 2a and b). Time-dependent uptake of [^18^F]**21** demonstrated a rapid cellular uptake (10 min: 60.18% ± 3.84%), and the uptake maintained at high levels after incubation for 1 h. The uptake of [^177^Lu]**21** in HT-1080-FAP cells decreased slowly from 1 h (72.53% ± 3.43%) to 24 h (45.06% ± 2.62%). Efflux experiments (Fig. 2c and d) suggested that [^177^Lu]**21** demonstrated slower excretion up to 4 h than [^177^Lu]Lu-FAPI-04. Furthermore, both [^18^F]**21**/[^177^Lu]**21** and [^68^ Ga]/[^177^Lu]Ga/Lu-FAPI-04 showed rapid internalization rate (10 min: > 80%), while [^18^F]**21**/[^177^Lu]**21** exhibited slightly higher internalization than corresponding [^68^ Ga]/[^177^Lu]Ga/Lu-FAPI-04 (Fig. 2e and f).

### Micro PET/CT imaging

**Fig. 3:**
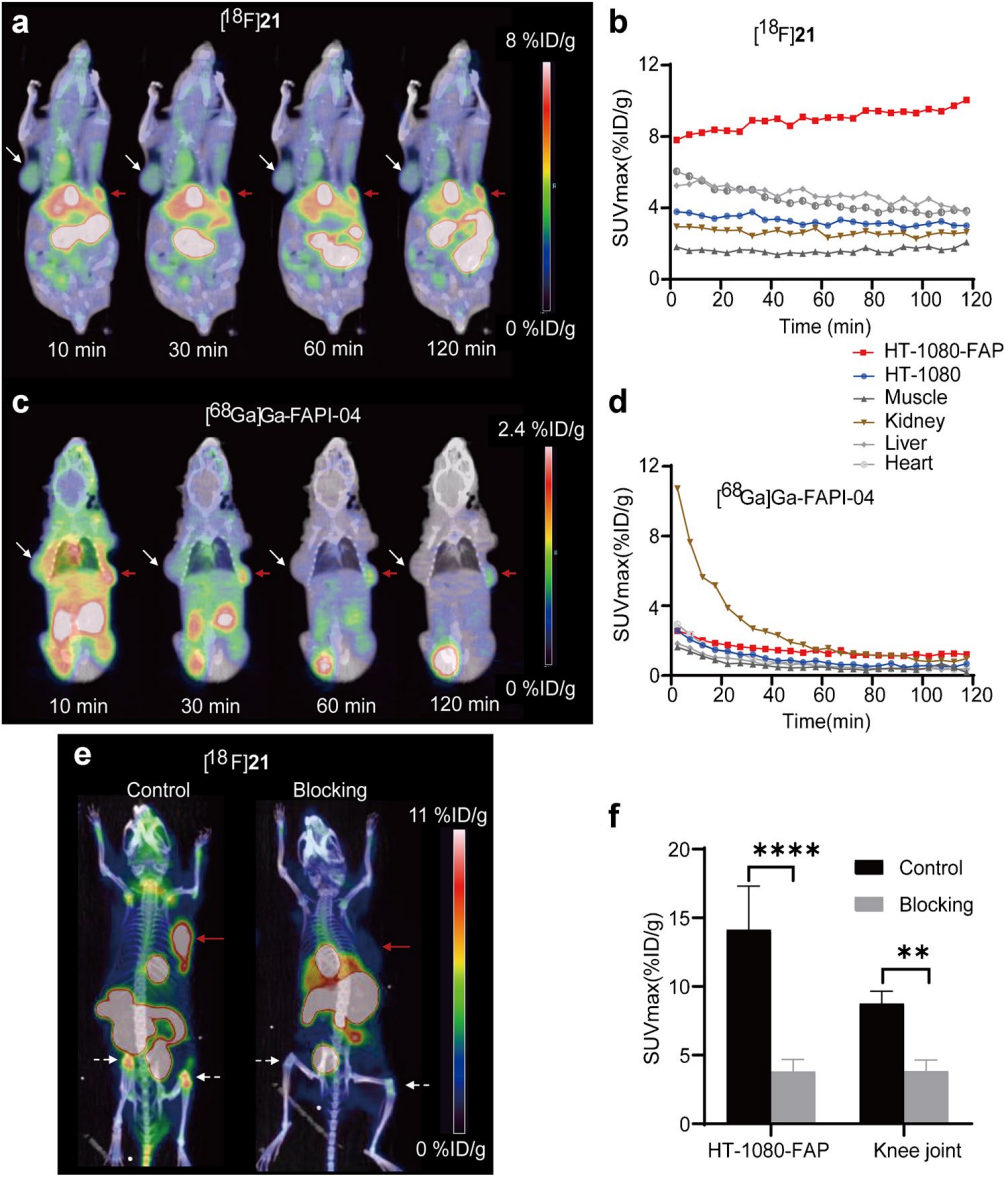
Dynamic PET imaging of [^18^F]**21** (**a**) and [^68^ Ga]Ga-FAPI-04 (**c**) in the same HT-1080-FAP and HT-1080 tumor-bearing mouse. Corresponding time–activity curves for tumors and major organs of [^18^F]**21** (**b**) and [^68^ Ga]Ga-FAPI-04 (**d**). [^18^F]**21** PET imaging (**e**) with and without the competitor FAPI-04. The corresponding ROI analysis (**f**) of tumor and knee joints uptake. Red arrow = HT-1080-FAP xenograft; White arrow = HT-1080 xenograft; White dotes arrow = knee joint. **P < 0.01. ****P < 0.0001

A comparison of dynamic micro-PET/CT imaging with [^18^F]**21** and [^68^ Ga]Ga-FAPI-04 was performed in the same mice bearing HT-1080 and HT-1080-FAP xenografts to evaluate the pharmacokinetics and FAP specificity (Fig. 3a–d). The time-activity curves (Fig. 3b and d) showed both radiotracers were rapidly accumulated in HT-1080-FAP tumor, and FAP-negative HT-1080 tumors did not show any uptakes. More importantly, HT-1080-FAP tumor uptake of [^18^F]**21** was increased linearly up, while [^68^ Ga]Ga-FAPI-04 showed a constantly decreasing tumor uptake over the total scan time, resulting in 14.9-fold higher tumor uptake for [^18^F]**21** than [^68^ Ga]Ga-FAPI-04 at 2 h pi (10.04%ID/g vs. 0.673%ID/g). Moreover, uptakes of [^18^F]**21** was higher in background, liver, gallbladder and intestine, suggesting hepatobiliary excretion due to its lipophilicity. The blocking studies of [^18^F]**21** with FAPI-04 (Fig. 3e–f) showed a significant reduction in tumor uptake (14.13 ± 3.19%ID/g vs. 3.8 ± 0.89%ID/g), validating the FAP specificity of [^18^F]**21** in vivo (Fig. 3e–f). Notably, the uptake in joints was also blocked significantly.

### SPECT imaging

**Fig. 4:**
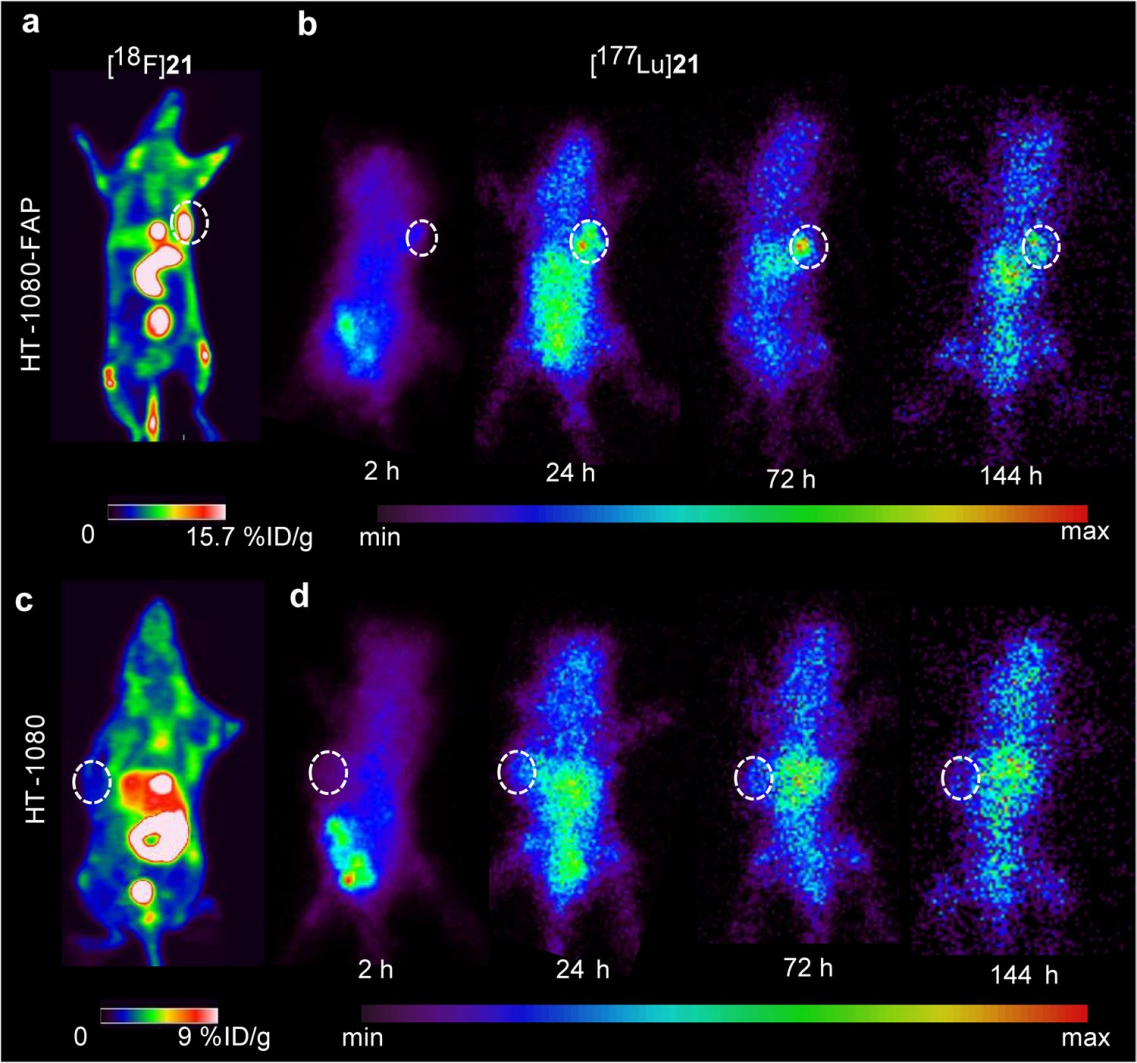
The consistent positive uptake of HT-1080-FAP xenograft in static PET imaging of [^18^F]**21** (**a**) and in SPECT imaging of [^177^Lu]**21** (**b**) in the same mouse. The consistent negative uptake of HT-1080 xenograft in PET imaging of [^18^F]**21** (**c**) and in SPECT imaging of [^177^Lu]**21** (**d**) at various time points in the same mouse. The circles represent the corresponding tumor

SPECT imaging of [^177^Lu]**21** was performed to investigate the tumor retention over a long time scale. For comparison, the same mouse underwent a [^18^F]**21** PET scan before SPECT imaging. As expected, both tracers displayed high uptake in HT-1080-FAP tumors (Fig. 4a and b) and low uptake in HT-1080 tumors (Fig. 4c and d). The results were similar to the previous micro-PET studies. In addition, high accumulation of [^177^Lu]**21** was observed in tumor-bearing mice up to 144 h pi, indicating a prolonged tumor retention. High radioactivity in abdominal regions was also noted.

### Biodistribution

**Fig. 5:**
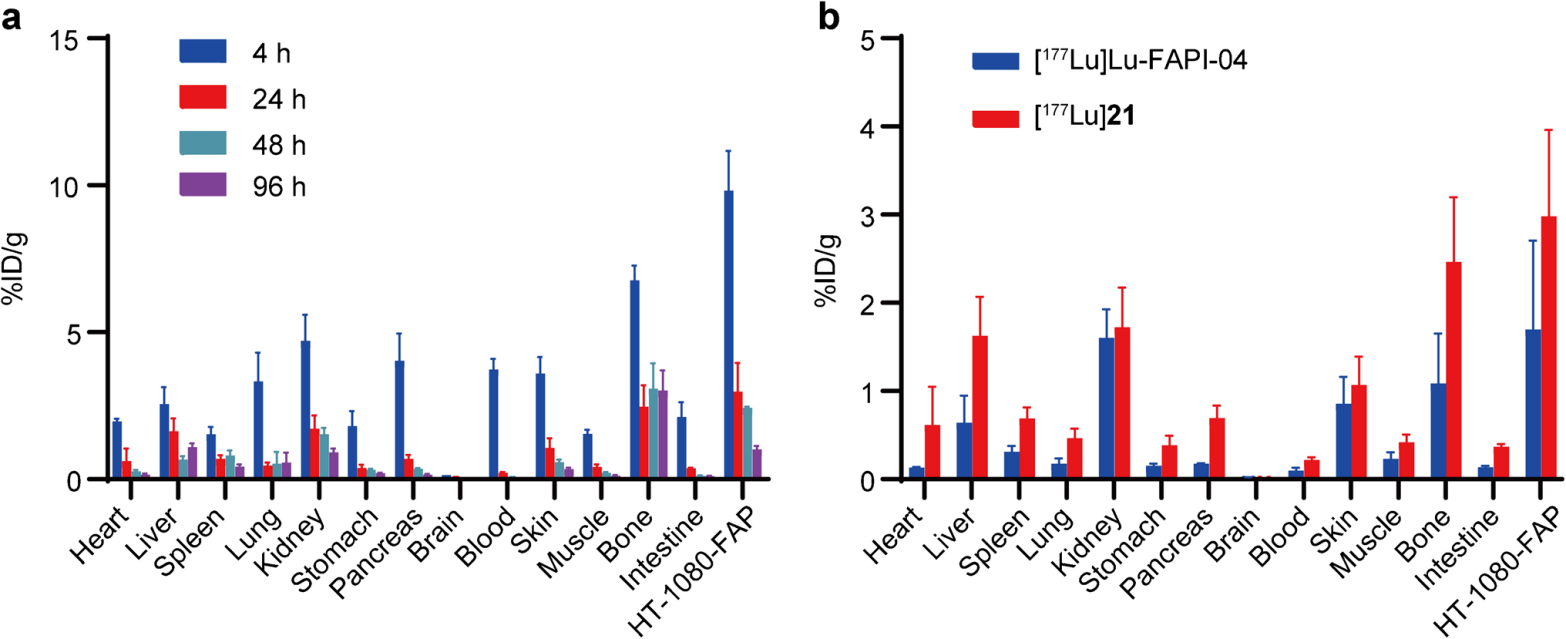
Biodistribution of ^177^Lu-labeled FAPI radioligands in HT-1080-FAP tumor-bearing mice. **a** Biodistribution of [^177^Lu]**21** at different time points. **b** Comparison of [^177^Lu]**21** and [^177^Lu]Lu-FAPI-04 at 24 h pi. *n* = 3/group

Biodistribution data was presented in Fig. 5 and Supplementary Tables 1–2. [^177^Lu]**21** showed a distinct tumor uptake that peaked at 4 h pi (9.82 ± 1.35%ID/g) and gradually decreased to 1.02 ± 0.11%ID/g at 96 h pi. Low activity concentrations were observed in most tissues at 96 h pi. Notably, an increasing tumor/blood and tumor/muscle ratio was observed over time. The blood uptake of [^177^Lu]**21** decreased rapidly from 3.73 ± 0.36%ID/g to 0.01 ± 0.00%ID/g at 96 h pi, resulting in substantially high tumor/blood ratio (102.33 ± 11.24, Supplementary Fig. 2 and Table 2). Tumor-to-muscle ratio achieved maximum at 48 h pi with value of 11.31 ± 1.41. Furthermore, [^177^Lu]**21** (2.98 ± 0.98%ID/g) displayed a significantly higher tumor uptake than that of [^177^Lu]Lu-FAPI-04 (1.70 ± 1.01%ID/g), thus presenting a similar or better tumor/non-tumor ratio at 24 h pi.

### Radionuclide therapy

**Fig. 6:**
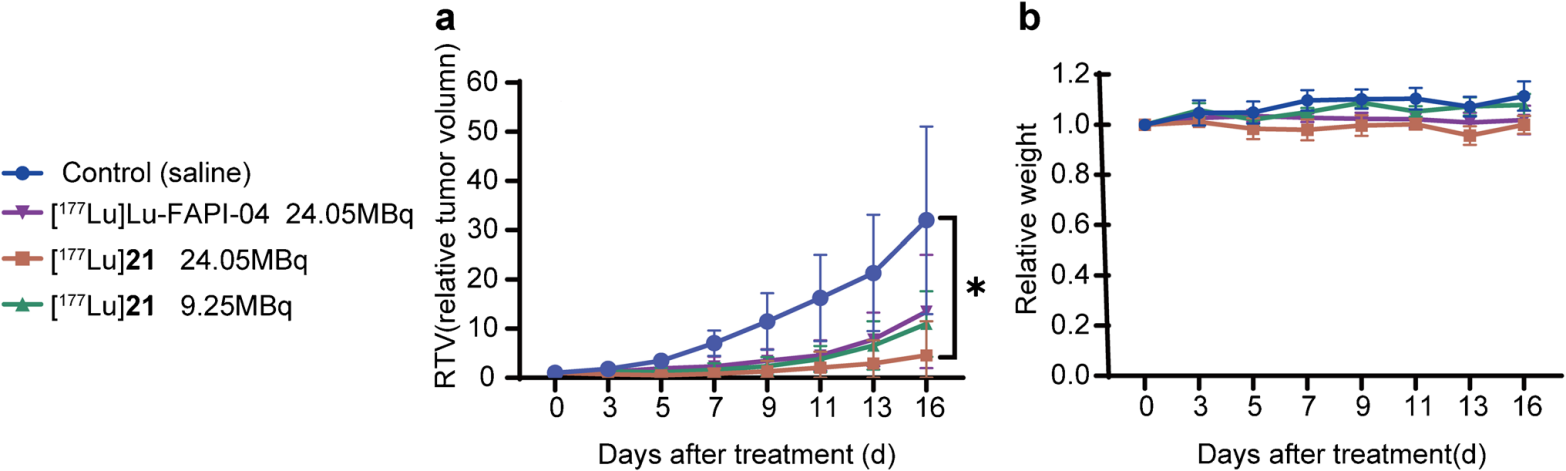
Radionuclide treatment study of [^177^Lu]**21** and [^177^Lu]Lu-FAPI-04 in HT-1080-FAP tumor-bearing mice(*n* = 4/group). Relative tumor volume (**a**), body weight change (**b**) after treatment. *P < 0.05

All treated groups exhibited significant anti-tumor activity compared with the control group. By the end of this study, tumor treated with 24.05 MBq of [^177^Lu]**21** were significantly smaller than those treated with 24.05 MBq of [^177^Lu]Lu-FAPI-04 (average relative tumor volume: 32.01 vs. 4.55; *P* = 0.033). In addition, the groups treated by 24.05 MBq of [^177^Lu]Lu-FAPI-04 and 9.25 MBq of [^177^Lu]**21** showed similar suppression of tumor growth, though both groups had no statistically significant difference in comparison to control (Fig. 6a), possibly due to the limited sample size. These results were consistent with high and prolonged retention HT-1080-FAP tumor uptake of [^177^Lu]**21**. The relative weight changed over time ranging from 0.92 to 1.17 (Fig. 6b). The pathology results indicated high FAP expression in HT-1080-FAP xenografts (Supplementary Fig. 3). The pathology results of major organ showed no significant difference between the control and ^177^Lu-treated groups (Supplementary Fig. 4). Further investigations will be conducted in the follow-up study to monitor possible side effects.

### Autoradiography and histology

**Fig. 7:**
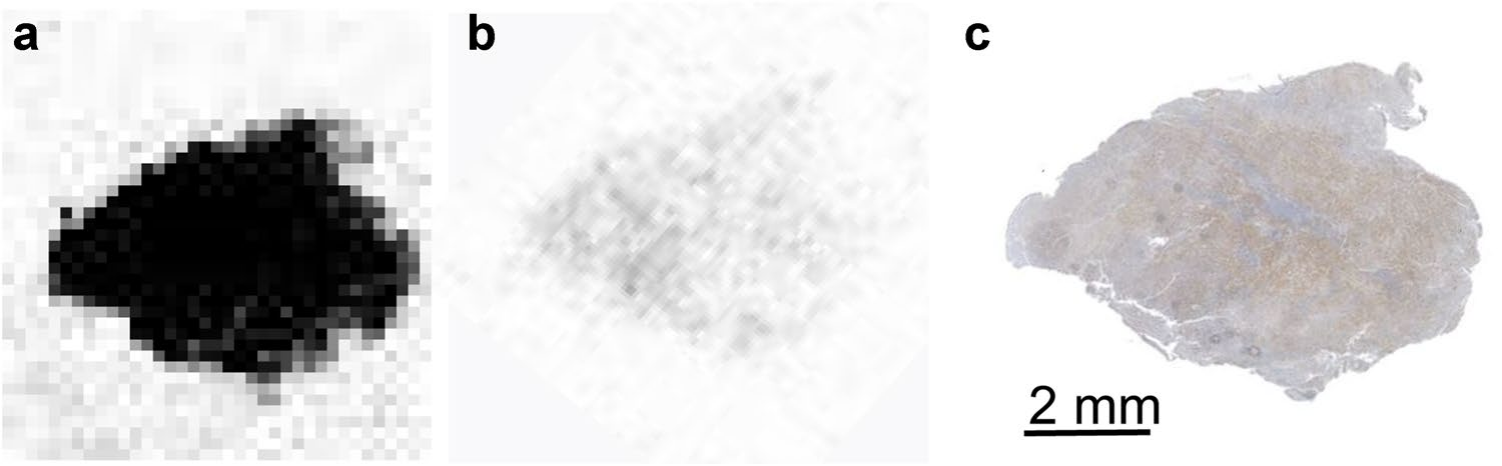
**a** In vitro autoradiography result of HT-1080-FAP tumor slide. **b** In vitro autoradiography result of HT-1080-FAP tumor slide with unlabeled **20** (130 μM). **c** IHC result of FAP expression of HT-1080-FAP tumor slide

In vitro autoradiography and histology results (Fig. 7) indicated that radioactivity enriched area was relevant to FAP-positive area in histology, which could be blocked by unlabeled **20**.

## Discussion

Recently, FAP has attracted extensive attention for its potential as a cancer imaging and therapeutic target. In this study, we combined SiFA moiety and a chelator, DOTAGA, in a single molecule in order to develop a FAP-targeted radiohybrid theranostics agent with excellent properties. The radiohybrid strategy could offer several advantages. Firstly, SiFA moiety enables highly efficient and robust fluorine-18 labeling that allows kit-like production without any complex procedures. Secondly, due to the high inherent lipophilicity of SiFA, ligands with SiFA moiety might show high lipophilicity, and be trapped and metabolized in the liver and intestine [25]. The lipophilicity will be significantly reduced by the addition of DOTAGA with four carboxylic acid group [20]. Thirdly, the SiFA and DOTAGA could be labeled with corresponding radio isotopes (fluorine-18 and lutetium-177) in an independent manner, and used for diagnostic imaging or radiotherapy. More importantly, the resulting [^18^F]**21** and [^177^Lu]**21** shared chemically identical molecule, thus presenting almost identical biodistribution in human bodies, whereas the widely reported theranostics approach with a pair of radio isotopes, gallium-68 and lutetium-177, used different radiometal-complexes to serve diagnostic and therapeutic purpose [10, 11]. Such radiohybrid strategy combining fluorine-18 and lutetium-177 in one single molecule was likely to offer more accurate dosimetry predictions and facilitate pretherapeutic patient stratification.

Therefore, to take advantage of the favorable properties of fluorine-18 and realize the integration of diagnosis and treatment, we developed and evaluated a radiohybrid FAP-targeted ligand. [^18^F]**21** could be produced at r.t. in 20 min without azeotropically drying or HPLC purification. The highly efficient ^18^F-^19^F isotopic exchange and simple purification procedure with SPE cartridge resulted in quick labeling reactions under mild conditions, which is more clinically applicable than widely used azeotropically drying procedures. Typically, the RCY and molar activity of SiFA-bearing ligands, such as [^18^F]SiTATE [26] and [^18^F]rhPSMA [20], was usually higher than 60% and 60 GBq/μmol, respectively. However, a relatively low RCY (22.9%) and molar activity (1.17–3.54 GBq/μmol) were obtained due to limited radioactivity (182.04–307.1 MBq) and precursor amount (30 μg) used in our initial experiments. Future optimization with high radioactivity (7400 MBq) and large amount of precursor (75 μg) significantly increased the RCY (61.8%) and molar activity (91.2–106 GBq/μmol).

The main purpose of this study was to evaluate the diagnostic and therapeutic efficacy of [^18^F]**21** and [^177^Lu]**21** in preclinical settings in comparison to the well-established agent [^68^ Ga]/[^177^Lu]Ga/Lu-FAPI-04. Both [^18^F]**21** and [^177^Lu]**21** showed high in vitro/in vivo stability (> 90%). Besides, [^18^F]**21**/[^177^Lu]**21** displayed a higher lipophilicity than [^68^ Ga]/[^177^Lu]Ga/Lu-FAPI-04. As we mentioned before, the relatively rapid washout from the circulation and tumor is still a limitation for the first generation of FAPI ligand [8, 9]. New tracers with higher lipophilicity might contribute to the improvement of pharmacokinetics. Given that SiFA exercised a profound lipophilic influence, additional combinations of hydrophilic auxiliaries, glycine and tranexamic acid, were introduced into the molecular structure to optimize the lipophilicity [27]. Furthermore, **20** showed excellent binding affinity (IC_50_: 2.29 nM) towards FAP, while the IC_50_ of FAPI-04 was 6.69 nM. A significantly higher cellular uptake of [^18^F]**21** was observed after 1 h incubation, compared to that of [^68^ Ga]Ga-FAPI-04 (86.43% ± 5.46% vs. 7.65% ± 1.0%), which might be attributed to high lipophilicity of **20**. In addition, the uptake could be blocked by UAMC1110, which indicated high binding specificity to FAP. The cellular internalization rates of [^18^F]**21** were consistently higher than those of [^68^ Ga]Ga-FAPI-04. As expected, similar results were observed for the cellular studies of [^177^Lu]**21** and [^177^Lu]Lu-FAPI-04.

We performed a head-to-head comparison of micro-PET/CT imaging between [^18^F]**21** and [^68^ Ga]Ga-FAPI-04 in the same tumor-bearing mouse. Our data demonstrated clear differences in tumor uptake between two tracers. [^18^F]**21** exhibited substantially higher HT-1080-FAP tumor uptake than [^68^ Ga]Ga-FAPI-04 at all assessed time points and the blocking studies led to a significant reduction in tumor uptake. Also, FAP-negative HT-1080 tumor-bearing mice revealed low tumor uptake. These results indicated high and specific binding to FAP in tumor. Similar to [^18^F]Glc-FAPI [17] and [^18^F]AlF-P-FAPI [19], the uptake of [^18^F]**21** was also observed in knee and shoulder joints, which could be blocked by unlabeled FAPI-04, suggesting FAP-specific uptake of joints. This finding agreed with a previous report [28]. More importantly, time-activity curves revealed rapid internalization and prolonged retention in HT-1080-FAP tumor, which might lead to high-quality PET images with significant improvement in tumor uptake. Therefore, we clearly demonstrated the feasibility of PET imaging of FAP-associated tumors with [^18^F]**21** as a novel imaging agent.

SPECT imaging and biodistribution studies were conducted to evaluate the pharmacokinetics of [^177^Lu]**21**. Prior to that, PET imaging studies with [^18^F]**21** were performed in the same mice. [^177^Lu]**21** demonstrated a similar distribution by SPECT as [^18^F]**21** with a significant and specific tumor uptake that could be visualized up to 6 days, indicating a prolonged retention in FAP-positive tumors. A head-to-head comparison with [^177^Lu]Lu-FAPI-04 showed that [^177^Lu]**21** exhibited higher levels and longer retention time within tumors at 24 h pi, and thus offered more prominent therapeutic efficacy than [^177^Lu]Lu-FAPI-04. These results are consistent with the preclinical data described above, e.g. better binding assay against FAP and higher cellular uptake. The uptakes of [^177^Lu]**21** in other tissues were also higher than that of [^177^Lu]Lu-FAPI-04. Indeed, [^177^Lu]**21** with relatively high lipophilicity was mainly cleared via hepatobiliary excretion, leading to increasing accumulation in the liver and digestive tract, which should be noted for its possible toxicity in radionuclide therapy. Therefore, H&E staining study in major organs at the end of treatments was performed, and no obvious tissue damage and adverse effect to these organs could be observed in the control and treated groups. The therapeutic efficacy of [^177^Lu]**21** in tumor-bearing mice was compared to [^177^Lu]Lu-FAPI-04. In line with increased tumor uptake and prolonged tumor retention, the tumor growth inhibition of [^177^Lu]**21** was much better than [^177^Lu]Lu-FAPI-04 after treatment at the same activity level (24.05 MBq). Given the relatively high uptake in normal tissues, the treatment with a reduced does of [^177^Lu]**21** (9.25 MBq) was evaluated as well, and it showed reduced toxicity and slightly better control of established tumors compared to [^177^Lu]Lu-FAPI-04 (24.05 MBq).

There are limitations in our study. Firstly, the resolution of [^177^Lu]**21** image was poor, because the scan was performed in Symbia Intevo Bold SPECT/CT system. Further SPECT imaging in a micro-SPECT system with practical high-resolution is warranted. Secondly, the SPECT imaging of [^177^Lu]Lu-FAPI-04 should be performed for further comparison. Thirdly, radionuclide therapy with larger sample size is needed to further evaluate the potential therapeutic efficacy.

## Conclusion

By combining SiFA and DOTAGA chelator in single molecule, we have successfully prepared and evaluated a novel radiohybrid theranostics ligand targeting FAP. The highly efficient and robust fluorine-18/lutetium-177 labeling allow kit-like production without any complex procedures. Compared with [^68^ Ga]/[^177^Lu]Ga/Lu-FAPI-04, [^18^F]**21**/[^177^Lu]**21** displayed significantly higher cellular uptake, better binding affinity, higher uptake and longer retention in HT-1080-FAP xenograft, resulting in high promising imaging properties and substantial therapeutic efficacy. The radiohybrid strategy, exploiting SiFA and DOTAGA chelator, may serve as useful platforms to design and create theranostics ligands for treatment of FAP-positive tumor.

## Supplementary Information

Below is the link to the electronic supplementary material.

Supplementary file1 (DOC 19584 KB)

## Data Availability

All data generated or analyzed during this study are included in this published article and its supplementary information file.
