# Supplementary material for: A radiohybrid theranostics ligand labeled with fluorine-18 and lutetium-177 for fibroblast activation protein-targeted imaging and radionuclide therapy
Source: Eur J Nucl Med Mol Imaging. 2023 Mar 3;50(8):2331–41. doi: 10.1007/s00259-023-06169-5 (PMC10250256; doi:10.1007/s00259-023-06169-5)
Supplement: Supplementary file 1 — Supplementary file1 (DOC 19584 KB) [file 259_2023_6169_MOESM1_ESM.doc]

Supporting Information For

**A Radiohybrid Theranostics Ligand Labeled with Fluorine-18 and Lutetium-177 for Fibroblast Activation Protein-targeted Imaging and Radionuclide Therapy**

Tianhong Yang*, Lei Peng*, Jia Qiu*, Xingjin He, Dake Zhang, Renbo Wu, Jianbo Liu, Xiangsong Zhang†, Zhihao Zha†

**Journal name:** European Journal of Nuclear Medicine and Molecular Imaging

**Affiliation for all authors:** Department of Nuclear Medicine, The First Affiliated Hospital of Sun Yat-sen University, 58# Zhongshan Er Road, Guangzhou, 510080, Guangdong Province, China.

†**Address correspondence to:** Zhihao Zha (zhazhh@mail.sysu.edu.cn; Tel: +8617319488127) and Xiangsong Zhang ([zhxiangs@mail.sysu.edu.cn](mailto:zhxiangs@mail.sysu.edu.cn); +8613711471890).

Contents

[1. Synthesis route of LuFL 3](#__RefHeading___Toc125389306)

[1.1 General procedure for preparation of compound 13 4](#__RefHeading___Toc125389307)

[1.2 General procedure for preparation of compound 17 4](#__RefHeading___Toc125389308)

[1.3 General procedure for preparation of compound 18 5](#__RefHeading___Toc125389309)

[1.4 General procedure for preparation of compound 19 5](#__RefHeading___Toc125389310)

[1.5 General procedure for preparation of compound 20, LuFL 6](#__RefHeading___Toc125389311)

[1.6 General procedure for preparation of compound 21, [^nat^Lu]Lu-LuFL 6](#__RefHeading___Toc125389312)

[2. Results of in vitro/in vivo stability analysis 7](#__RefHeading___Toc125389313)

[3. Results of tumor/normal tissues ratio in biodistribution study 7](#__RefHeading___Toc125389314)

[4. IHC staining results of FAP in HT-1080-FAP xenograft 8](#__RefHeading___Toc125389315)

[5. H&E staining results of main organs 8](#__RefHeading___Toc125389316)

[6. Data of biodistribution and tumor/normal tissues ratio 9](#__RefHeading___Toc125389317)

[References 10](#__RefHeading___Toc125389318)

# Synthesis route of LuFL


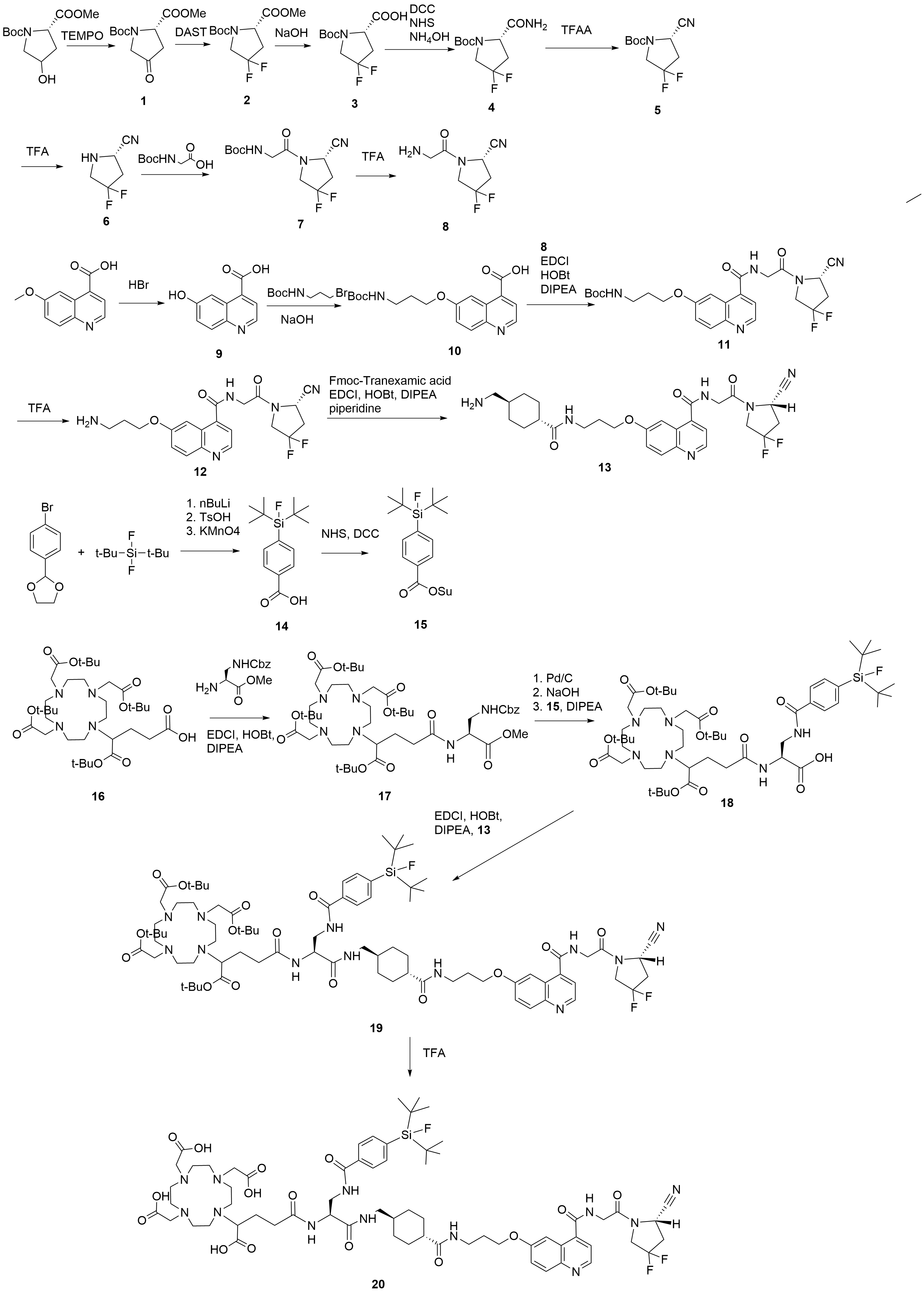


**Scheme. 1** Synthesis of LuFL

**Scheme. 1** depicts the synthesis route of LuFL. Compound **1**-**12**, **14, 15** and **16** are synthesized as previously reported [1-3].

## 1.1 General procedure for preparation of compound 13

**6-(3-((1r,4r)-4-(Aminomethyl)cyclohexane-1-carboxamido)propoxy)-N-(2-((S)-2-cyano-4,4-difluoropyrrolidin-1-yl)-2-oxoethyl)quinoline-4-carboxamide (13).** To a solution of **12** (208.5 mg，0.5 mmol) in DMF (10 mL), *N,N*-diisopropylethylamine (DIPEA, 129 mg, 1 mmol), 1-hydroxybenzotriazole hydrate (HOBt, 84.4 mg, 0.5 mmol), O-(1H-Benzotriazol-1-yl)-N,N,N',N'-tetramethyluronium hexafluorophosphate (HBTU, 37 mg, 0.19 mmol) and Fmoc-tranexamic acid (190 mg, 0.5 mmol) were added at 0 °C. After the mixture was stirred at room temperature (r.t.) overnight, piperidine (2 mL) was added. The resulting mixture was stirred at r.t. for 3 h before 50 mL of EtOAc was added to the reaction mixture. It was then washed with H_2_O (15 mL × 2) and brine (15 mL), dried over MgSO_4_, and filtered. The filtrate was concentrated, and the residue was purified by flash chromatography (FC) (DCM/MeOH/NH_4_OH = 90/9/1) to give 172 mg **13** as colorless oil (yield: 62.1%): ^1^HNMR (400 MHz, CDCl_3_) δ: 8.84 (d, 1H, *J* = 4.3 Hz), 8.06 (d, 1H, *J* = 9.2 Hz), 7.71 (d, 1H, *J* = 2.8 Hz), 7.73 (d, 1H, *J* = 4.3 Hz), 7.40 (dd, 1H, *J* = 2.8 Hz, 9.2 Hz), 7.11 (br, s, 1H), 5.91 (br, s, 1H), 5.04-5.07 (m, 1H), 4.40-4.46 (m, 1H), 4.21-4.31 (m, 3H), 4.02-4.15 (m, 3H), 2.81-2.90 (m, 3H), 2.64-2.70 (m, 1H), 2.07-2.10 (m, 2H), 1.97-2.04 (m, 1H), 1.84-1.93 (m, 4H), 1.02-1.56 (m, 5H). HRMS calcd for C_28_H_35_F_2_N_6_O_4_ (M + H)^+^, 557.2688; found 557.2697.

## 1.2 General procedure for preparation of compound 17

**Tri-tert-butyl 2,2',2''-(10-((6S)-6-(methoxycarbonyl)-14,14-dimethyl-3,8,12-trioxo-1-phenyl-2,13-dioxa-4,7-diazapentadecan-11-yl)-1,4,7,10-tetraazacyclododecane-1,4,7-triyl)triacetate (17).** To a solution of **16** (700 mg, 1 mmol) in DMF (15 mL), DIPEA (774 mg, 6 mmol), HOBt (253 mg, 1.5 mmol), *N*-(3-dimethylaminopropyl)-*N*-ethylcarbodiimide hydrochloride (EDCI, 286 mg, 1.5 mmol) and (S)-Methyl 2-amino-3-(((benzyloxy)carbonyl)amino)propanoate hydrochloride (289 mg, 1 mmol) were added at 0 °C. The resulting mixture was stirred at r.t. for overnight before 50 mL of EtOAc was added to the reaction mixture. It was then washed with H_2_O (15 mL × 2) and brine (15 mL), dried over MgSO_4_, and filtered. The filtrate was concentrated, and the residue was purified by flash chromatography (FC) (DCM/MeOH/NH_4_OH = 90/9/1) to give 344 mg **17** as colorless oil (yield: 74.8%): ^1^HNMR (400 MHz, CDCl_3_) δ: 8.98 (dd, 1H, J = 5.4 Hz, 19.9 Hz), 7.20-7.36 (m, 5H), 5.06 (s, 2H), 4.40-4.42 (m, 1H), 3.05-3.76 (m, 7H), 3.45 (s, 3H), 3.27-3.38 (m, 2H), 2.82-2.99 (m, 4H), 2.68-2.73 (m, 6H), 2.48-2.52 (m, 2H), 2.23-2.29 (m, 4H), 2.05-2.14 (m, 3H), 1.80-1.85 (m, 1H), 1.44 (s, 36H). HRMS calcd for C_47_H_79_N_6_O_13_ (M + H)^+^, 935.5705; found 935.5715.

## 1.3 General procedure for preparation of compound 18

**(2S)-2-(5-(tert-Butoxy)-5-oxo-4-(4,7,10-tris(2-(tert-butoxy)-2-oxoethyl)-1,4,7,10-tetraazacyclododecan-1-yl)pentanamido)-3-(4-(di-tert-butylfluorosilyl)benzamido)propanoic acid (18).** To a solution of **17** (186 mg, 0.2 mmol) in MeOH (10 mL) was added Pd/C (10%, 20 mg). The pressure of reaction flask was reduced with a vacuum pump and H_2_ was backfilled using a balloon. The reaction was stirred at r.t. with H_2_ for overnight. Pd/C was then filtered and the solvent was removed by vacuum pump. A solution of NaOH in EtOH/H_2_O (1 M, 1/1, 5 mL) was added to the residue and maintained at r.t. for 2 h. The mixture was cooled with ice-bath and HCl (1 M) was added dropwise till pH = 7. EtOAc (30 mL) was added to the mixture and the organic phase was washed with H_2_O (10 mL × 2) and brine (10 mL), dried over MgSO_4_, and filtered. The filtrate was concentrated. DCM (5 mL) was added to the residue, followed by DIPEA (51.6 mg, 0.4 mmol) and **15** (75.8 mg, 0.2 mmol). The reaction mixture was then stirred at r.t. overnight and the solvent was removed, and the residue was purified by FC (DCM/MeOH/NH_4_OH = 80/20/2) to give 86 mg **18** as colorless oil (yield: 40.6%): ^1^HNMR (400 MHz, CDCl_3_) δ: 7.84-7.87 (m, 2H), 7.72-7.74 (m, 2H), 4.46-4.48 (m, 1H), 3.71-3.85 (m, 2H), 3.45-3.54 (m, 4H), 3.07-3.14 (m, 3H), 2.78-2.95 (m, 4H), 2.63-2.68 (m, 6H), 2.43-2.47 (m, 2H), 2.18-2.24 (m, 4H), 2.00-2.09 (m, 3H), 1.75-1.80 (m, 1H), 1.50 (s, 36H), 1.09 (s, 18H). HRMS calcd for C_53_H_22_FN_6_O_12_Si (M + H)^+^, 1051.6527; found 1051.6539.

## 1.4 General procedure for preparation of compound 19

**Tri-tert-butyl 2,2',2''-(10-(1-(tert-butoxy)-5-(((2S)-1-(((4-((3-((4-((2-((S)-2-cyano-4,4-difluoropyrrolidin-1-yl)-2-oxoethyl)carbamoyl)quinolin-6-yl)oxy)propyl)carbamoyl)cyclohexyl)methyl)amino)-3-(4-(di-tert-butylfluorosilyl)benzamido)-1-oxopropan-2-yl)amino)-1,5-dioxopentan-2-yl)-1,4,7,10-tetraazacyclododecane-1,4,7-triyl)triacetate (19).** To a solution of **18** (100 mg，0.1 mmol) in DMF (5 mL), DIPEA (25.8 mg, 0.2 mmol), HOBt (26.2 mg, 0.15 mmol), EDCI (25.8 mg, 0.15 mmol) and **13** (57.8 mg, 0.1 mmol) were added at 0 °C. The resulting mixture was stirred at r.t. for overnight before EtOAc (30 mL) was added to the reaction mixture. It was then washed with H_2_O (10 mL × 2) and brine (10 mL), dried over MgSO_4_, and filtered. The filtrate was concentrated, and the residue was purified by FC (DCM/MeOH/NH_4_OH = 90/9/1) to give 30 mg **19** as colorless oil (yield: 18.1%): ^1^HNMR (400 MHz, MeOD) δ: 8.75 (d, 1H, *J* = 4.3 Hz), 7.87-7.99 (m, 4H), 7.72-7.74 (m, 2H), 7.59 (d, 1H, *J* = 4.3 Hz), 7.49 (d, 1H, *J* = 9.2 Hz), 5.17 (d, 1H, *J* = 9.0 Hz), 4.01-4.41 (m, 8H), 3.37-3.53 (m, 6H), 2.45-3.17 (m, 14H), 2.03-2.38 (m, 13H), 1.79-1.95 (m, 3H), 1.54-1.66 (m, 4H), 1.23-1.47 (m, 41H), 1.08 (s, 18H). HRMS calcd for C_81_H_124_F_3_N_12_O_15_Si (M + H)^+^, 1589.9030; found 1589.9045.

## 1.5 General procedure for preparation of compound 20, LuFL

**2,2',2''-(10-(1-Carboxy-4-(((S)-1-((((1r,4S)-4-((3-((4-((2-((S)-2-cyano-4,4-difluoropyrrolidin-1-yl)-2-oxoethyl)carbamoyl)quinolin-6-yl)oxy)propyl)carbamoyl)cyclohexyl)methyl)amino)-3-(4-(di-tert-butylfluorosilyl)benzamido)-1-oxopropan-2-yl)amino)-4-oxobutyl)-1,4,7,10-tetraazacyclododecane-1,4,7-triyl)triacetic acid (20, LuFL).** A solution of **19** (30 mg, 0.018 mmol) in TFA (3 mL) was stirred at r.t. for 5 h. The reaction mixture was evaporated in vacuo, and the residue was recrystallized from Ether/EtOH. The resulting white solid was dissolved in MeOH (1 mL) and purified by semi-prep HPLC to give 13.6 mg **20** as a white solid (yield: 54.2%): ^1^HNMR (400 MHz, MeOD) δ: 8.98 (d, 1H, J = 3.9 Hz), 8.17-8.19 (m, 2H), 7.92 (d, 1H, J = 3.9 Hz), 7.84 (d, 2H. J = 7.6 Hz), 7.73-7.74 (m, 3H), 5.17 (d, 1H, J = 8.2 Hz), 4.23-4.41 (m, 8H), 4.10-4.20 (m, 2H), 3.74-4.06 (br, s, 8H), 3.15-3.53 (m, 18H), 2.83-3.02 (m, 4H), 2.67 (s, 4H), 2.38-2.41 (m, 2H), 2.06-2.11 (m, 4H), 1.84-1.95 (m, 2H), 1.54-1.71 (m, 4H), 1.38-1.46 (m, 2H), 1.04-1.12 (m, 20H). HRMS calcd for C_65_H_92_F_3_N_12_O_15_Si (M + H)^+^, 1365.6528; found 1365.6544.

## 1.6 General procedure for preparation of compound 21, [^nat^Lu]Lu-LuFL

**Compound** 21, **[^nat^Lu]Lu-LuFL**. Compound **20** (15 mg, 0.011 mmol) was dissolved in 0.4 mL of MeCN and 1.6 mL of water in a small vial, and 11 μL of [^nat^Lu]LuCl_3_ (1 M) in 0.05 N HCl was added. The mixture was carefully adjusted by dropwise addition of NaOAc (100 mg/mL) to pH = 5. The vial was closed, and the reaction mixture was stirred for 2 h at 95 °C. The solvent was removed and the residue was purified by semi-prep HPLC to give 12.6 mg **compound** 21, **[^nat^Lu]Lu-LuFL** as a white solid (yield: 74.5%): HRMS calcd for C_65_H_89_F_3Lu_N_12_O_15_Si (M + H)^+^, 1537.5699; found 1537.5613.

# 2. Results of in vitro/in vivo stability analysis


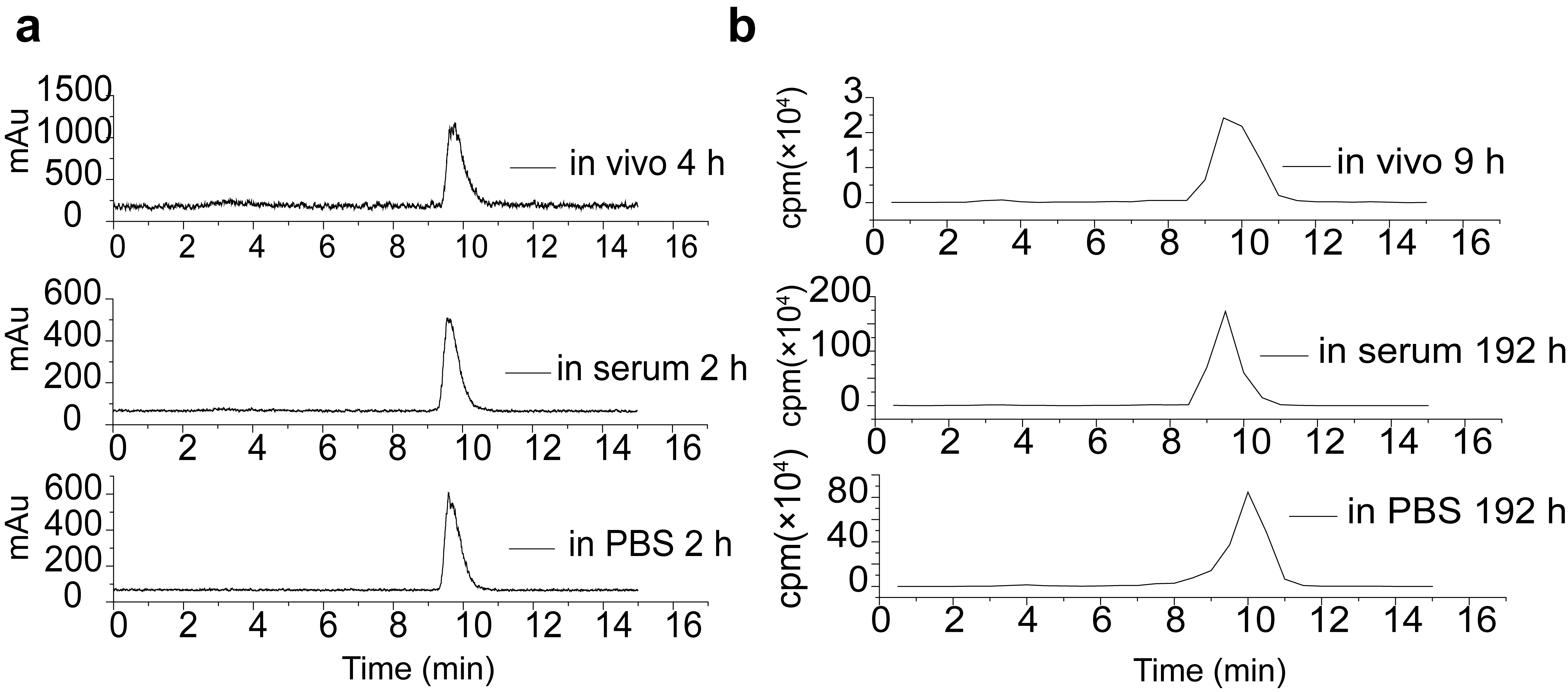


**Supplemental Fig. 1** In vitro and in vivo stability of [^18^F]**21** (**a**) and [^177^Lu]**21** (**b**)

# 3. Results of tumor/normal tissues ratio in biodistribution study


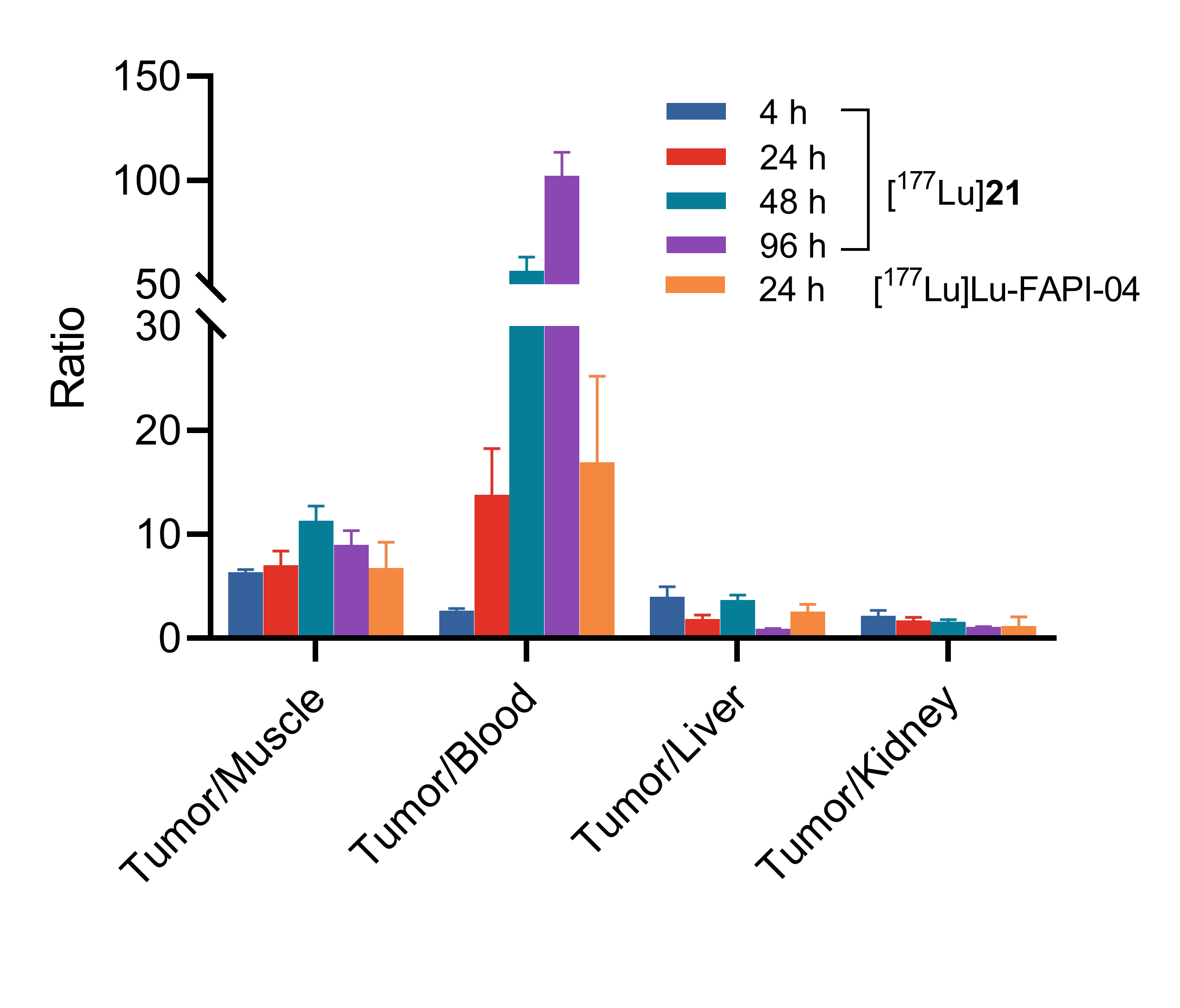


**Supplemental Fig. 2** Tumor/muscle, tumor/blood, tumor/liver and tumor/kidney ratio of [^177^Lu]**21** at 4, 24, 48, 96 h pi and [^177^Lu]Lu-FAPI-04 at 24 h pi in biodistribution study

# 4. IHC staining results of FAP in HT-1080-FAP xenograft


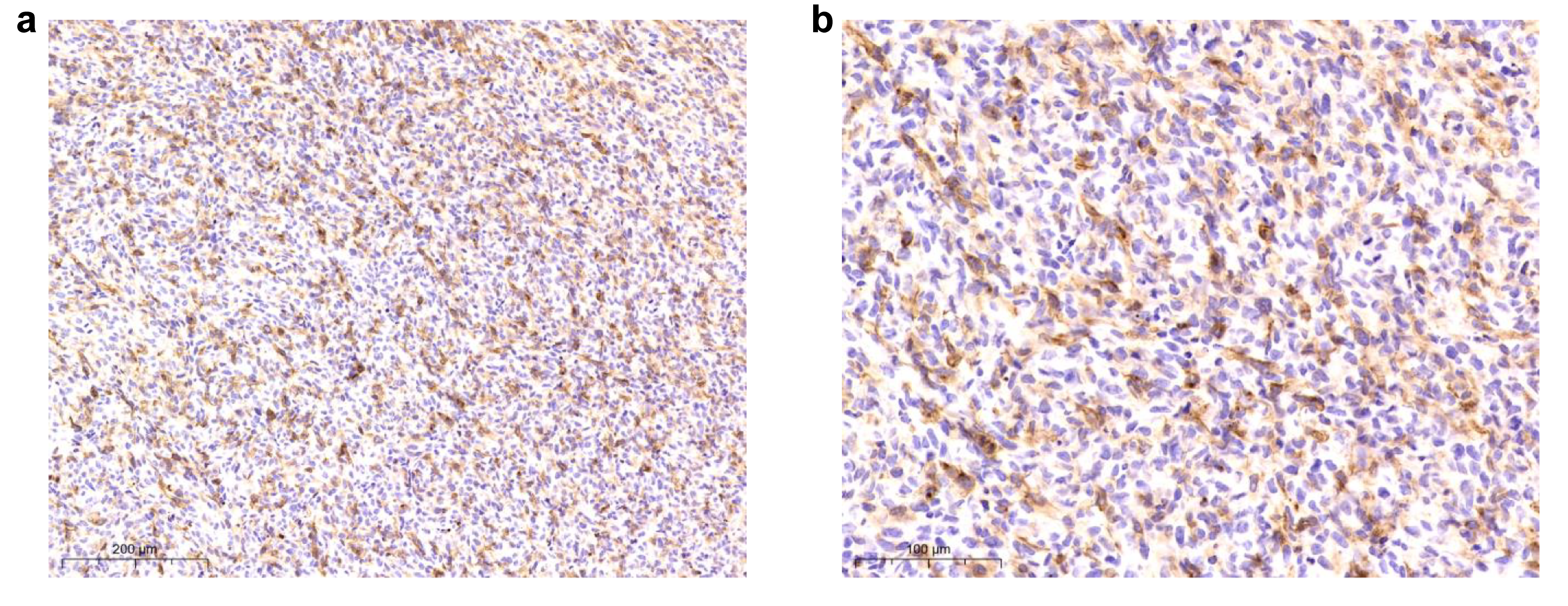


**Supplemental Fig. 3** IHC staining of FAP expression in HT-1080-FAP xenograft. Original magnification 20 x (**a**) and 40 x (**b**)

# 5. H&E staining results of main organs


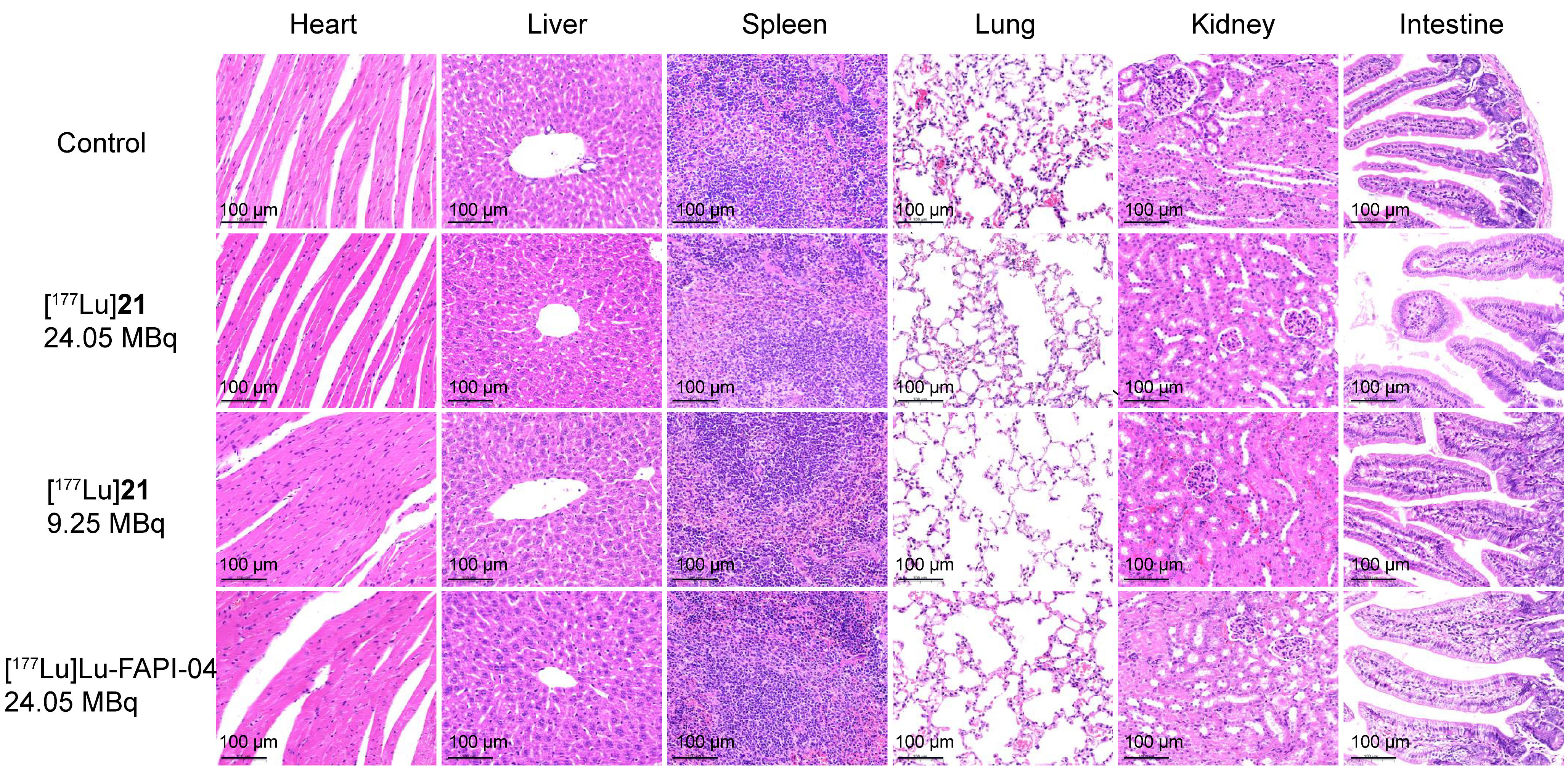


**Supplemental Fig. 4** H&E staining of main organs including heart, liver, spleen, lung, kidney and intestine after treatment with 24.05 MBq of [^177^Lu]**21**, 9.25 MBq of [^177^Lu]**21**, 24.05 MBq of [^177^Lu]Lu-FAPI-04 and saline, respectively

# 6. Data of biodistribution and tumor/normal tissues ratio

**Supplemental Table. 1** Biodistribution After Intravenous Administration of [^177^Lu]**21** at 4, 24, 48, 96 h pi and [^177^Lu]Lu-FAPI-04 at 24 h pi in HT-1080-FAP Xenograft Model

|  | [^177^Lu]**21** | | | | [^177^Lu]Lu-FAPI-04 |
| --- | --- | --- | --- | --- | --- |
|  | 4 h | 24 h | 48 h | 96 h | 24 h |
| heart | 1.97 ± 0.09 | 0.61 ±0.43 | 0.27 ± 0.05 | 0.16 ± 0.03 | 0.13 ± 0.01 |
| liver | 2.56 ± 0.57 | 1.63 ± 0.44 | 0.67 ± 0.11 | 1.10 ± 0.12 | 0.64 ± 0.31 |
| spleen | 1.53 ± 0.25 | 0.69 ± 0.13 | 0.81 ± 0.18 | 0.43 ± 0.07 | 0.31 ± 0.07 |
| lung | 3.33 ± 0.97 | 0.46 ± 0.11 | 0.53 ± 0.4 | 0.57 ± 0.34 | 0.18 ± 0.06 |
| kidney | 4.70 ± 0.89 | 1.72 ± 0.45 | 1.53 ± 0.21 | 0.92 ± 0.13 | 1.60 ± 0.33 |
| stomach | 1.80 ± 0.52 | 0.39 ± 0.11 | 0.32 ± 0.04 | 0.20 ± 0.01 | 0.15 ± 0.03 |
| pancreas | 4.03 ± 0.93 | 0.69 ± 0.14 | 0.35 ± 0.04 | 0.15 ± 0.03 | 0.18 ± 0.01 |
| brain | 0.14 ± 0.01 | 0.02 ± 0.00 | 0.00 ± 0.01 | 0.01 ± 0.00 | 0.02 ± 0.01 |
| blood | 3.73 ± 0.36 | 0.22 ± 0.03 | 0.04 ± 0.01 | 0.01 ± 0.00 | 0.10 ± 0.04 |
| skin | 3.60 ± 0.56 | 1.07 ± 0.32 | 0.58 ± 0.09 | 0.35 ± 0.05 | 0.86 ± 0.31 |
| muscle | 1.54 ± 0.14 | 0.42 ± 0.09 | 0.22 ± 0.03 | 0.12 ± 0.03 | 0.23 ± 0.07 |
| bone | 6.76 ± 0.51 | 2.46 ± 0.74 | 3.09 ± 0.86 | 3.02 ± 0.69 | 1.08 ± 0.57 |
| intestine | 2.12 ± 0.5 | 0.37 ± 0.03 | 0.12 ± 0.02 | 0.11 ± 0.01 | 0.14 ± 0.02 |
| HT-1080-FAP | 9.82 ± 1.35 | 2.98 ± 0.98 | 2.43 ± 0.04 | 1.02 ± 0.11 | 1.70 ± 1.01 |
| Data are mean ± SD (*n* = 3) | | | | | |

**Supplemental Table. 2** Tumor/Normal tissues Ratio After Intravenous Administration of [^177^Lu]**21** at 4, 24, 48, 96 h pi and [^177^Lu]Lu-FAPI-04 at 24 h pi in HT-1080-FAP Xenograft model

|  | [^177^Lu]**21** | | | | [^177^Lu]Lu-FAPI-04 |
| --- | --- | --- | --- | --- | --- |
|  | 4 h | 24 h | 48 h | 96 h | 24 h |
| Tumor/Muscle | 6.36 ± 0.27 | 7.03 ± 1.39 | 11.31 ± 1.41 | 9.0 ± 1.35 | 6.76 ± 2.47 |
| Tumor/Blood | 2.63 ± 0.23 | 13.77 ± 4.99 | 56.58 ± 6.63 | 102.33 ± 11.24 | 16.92 ± 8.27 |
| Tumor/Liver | 3.98 ± 1.00 | 1.84 ± 0.41 | 3.66 ± 0.52 | 0.93 ± 0.01 | 2.56 ± 0.73 |
| Tumor/ Kidney | 2.15 ± 0.55 | 1.73 ± 0.32 | 1.60 ± 0.19 | 1.11 ± 0.04 | 1.18 ± 0.89 |
| Data are mean ± SD（*n* = 3） | | | | | |

# References

1. Jansen K, Heirbaut L, Verkerk R, et al. Extended structure-activity relationship and pharmacokinetic investigation of (4-quinolinoyl)glycyl-2-cyanopyrrolidine inhibitors of fibroblast activation protein (FAP). *J Med Chem*. 2014;57:3053-3074.

2. Iovkova L, Wängler B, Schirrmacher E, et al. para-Functionalized aryl-di-tert-butylfluorosilanes as potential labeling synthons for ^18^F radiopharmaceuticals. *Chemistry - A European Journal*. 2009;15:2140-2147.

3. Abiraj K, Jaccard H, Kretzschmar M, Helm L, Maecke HR. Novel DOTA-based prochelator for divalent peptide vectorization: synthesis of dimeric bombesin analogues for multimodality tumor imaging and therapy. *Chem Commun*. 2008:3248-3250.
